# Supplementary material for: Stepwise Distributed Open Innovation Contests for Software Development: Acceleration of Genome-Wide Association Analysis
Source: Gigascience. 2017 Feb 28;6(5):1–10. doi: 10.1093/gigascience/gix009 (PMC5467032; doi:10.1093/gigascience/gix009)

Revised Manuscript
Click here to download Manuscript GigaScienceGWAS_SCE(1).docx

Stepwise Distributed Open Innovation Contests for Software Development - Acceleration of Genome-Wide Association Analysis

Andrew Hill[1], Po-Ru Loh[2], Ragu B. Bharadwaj[3,4], Pascal Pons[5], Jingbo Shang[6] Eva Guinan[7,8], Karim Lakhani[3,9,10], Iain Kilty[11] and Scott A. Jelinsky[11*]

[1]Research Business Technology, Pfizer Research, Cambridge, Massachusetts, USA,
Department of Epidemiology, Harvard T.H. Chan School of Public Health, Boston, Massachusetts, USA
[2] Department of Epidemiology, Harvard T.H. Chan School of Public Health, Boston, Massachusetts, USA Program in Medical and Population Genetics, Broad Institute of Harvard and MIT, Cambridge, Massachusetts, USA
[3]Babbage Analytic and Innovation, Boston Massachusetts, USA,
[4] Current affiliation, Nyrasta LLC
[5] Current affiliation, Criteo Labs, Paris, France
[6] Current affiliation, Computer Science Department, University of Illinois at Urbana-Champaign
[7]Harvard Medical School, Boston, Massachusetts, USA.
[8]Department of Radiation Oncology, Dana-Farber Cancer Institute, Boston, Massachusetts, USA.
[9]Harvard Business School, Boston, Massachusetts, USA.
[10]Harvard-NASA Tournament Lab, Institute for Quantitative Social Science.
[11]Department of Inflammation and Immunology, Pfizer Research, Cambridge, Massachusetts, USA,
*Corresponding Author

Email addresses:
AH: Andrew.Hill@pfizer.com
PRL: loh@hsph.harvard.edu
RBB: ragu@nyrasta.com
PP: p.pons@criteo.com
JS: shang7@illinois.edu
EG: Eva_Guinan@dfci.harvard.edu
KL: k@hbs.edu
IK: Iain.Kilty@pfizer.com
SAJ: Scott.Jelinsky@pfizer.com

* To whom correspondence should be addressed at:
Scott Jelinsky, Ph.D.
Pfizer Research
610 Main Street
Cambridge, MA 02140
Ph. 617-674-7272
Fax 845-474-5978

Abstract

Background:

The association of differing genotypes with disease related phenotypic traits offers great potential to both help identify new therapeutic targets and support stratification of patients who would gain the greatest benefit from specific drug classes. Development of low cost genotyping and sequencing has made collecting large scale genotyping data routine in population and therapeutic intervention studies. In addition, a range of new technologies are being used to capture numerous new and complex phenotypic descriptors. As a result, genotype and phenotype datasets have grown exponentially. Genome-wide association studies (GWAS) associate genotypes and phenotypes using methods such as logistic regression. As existing tools for association analysis limit the efficiency by which value can be extracted from increasing volumes of data, there is a pressing need for new software tools that can accelerate association analyses on large genotype-phenotype datasets.

Results:

Using open innovation (OI) and contest based crowdsourcing, the logistic regression analysis in a leading, community-standard genetics software package (PLINK 1.07) was substantially accelerated. OI allowed us to do this in less than 6 months by providing rapid access to highly skilled programmers with specialized, difficult-to-find skill sets. Through a crowd based contest a combination of computational, numeric and algorithmic approaches was identified that accelerated the logistic regression in PLINK 1.07 by 18- to 45-fold. Combining contest-derived logistic regression code with coarse-grained parallelization, multithreading, and associated changes to data initialization code further developed through distributed innovation, we achieved an end-to-end speedup of 591-fold for a data set size of 6678 subjects by 645863 variants, compared to PLINK 1.07's logistic regression. This represents a reduction in run time from 4.8 hours to 29 seconds. Accelerated logistic regression code developed in this project has been incorporated into the PLINK2 project.

Conclusions:

Using iterative competition based OI, we have developed a new, faster implementation of logistic regression for GWAS analysis. We present lessons learned and recommendations on running a successful OI process for bioinformatics.

Keywords

Open innovation, Crowdsourcing, Genome-wide association study, PLINK, Logistic regression

Background

Genome-wide association studies (GWAS) relate genetic variants in individuals with specific phenotypes such as disease status [1, 2]. GWAS have identified single nucleotide polymorphisms (SNPs), genes, biological pathways and networks underlying complex diseases, and have been applied to classify patients, predict drug response and define novel therapeutic potential [3, 4]. To have adequate statistical power, GWAS can require large numbers of individuals and polymorphic alleles, particularly for common, complex diseases in which multiple alleles contribute to disease risk and specific SNPs have small individual effects. Improved technology and decreasing costs have allowed routine collection of GWAS data from deeply phenotyped patient cohorts where many clinical traits beyond disease status are assessed. Correspondingly, the challenge has now shifted to the analysis of these large data sets, essentially shifting the bottleneck from data collection to data analysis, and motivating the development of new data analysis methods.

A number of software tools exist for analyzing genotype-phenotype associations. One of the most popular tools for analyzing GWAS results is the open source software PLINK[5], which provides a number of analysis functions, including logistic regression to associate genetic variants with binary phenotypes. However, for today’s large genotype-phenotype datasets, association analyses can take many hours for a single phenotype. A number of groups have described alternative algorithms and software for more rapid computation of associations between genetic variants and phenotypes, often motivated by detecting epistasis [6-10]. Our approach to this analytic challenge

was to accelerate PLINK's logistic regression function through OI and crowdsourcing competitions.

Crowdsourcing utilizes a diverse, external group of problem solvers with potentially varied knowledge bases and background to assist in addressing a well-defined question or problem. Open, prize-based contests allow motivated individuals to compete for cash prize(s) to solve the proposed problem, creating increased potential for new, innovative ideas and solutions and extreme value outcomes. While contestants generally compete for monetary prizes, there are other motivating factors including peer recognition, skill building, self –affirmation and perceived enjoyment that attract competitors. Over the last decade, OI approaches have been shown to regularly engage hundreds and sometimes thousands of problem-solvers to solve difficult and important science and technology problems. Utilization of crowdsourcing in life sciences [11-16] is now emerging as an important way to complement internal R&D efforts, as well as augment an organization's capacity for technical work. Here we describe the process and iterative strategy by which we have harnessed the power of crowdsourcing as applied to a complex analytic problem.

Methods

Figure 1 outlines our iterative approach using prize based crowdsourcing to speed up GWAS analysis. Our workflow started with profiling of the PLINK 1.07 application, and then proceeded via contest-based crowdsourcing to accelerate logistic regression. Faster logistic regression code was re-integrated into PLINK 1.07, and then donated back to the PLINK2 open-source project. In addition, we crowdsourced data input/output changes and multithreading work, and used coarse-grained parallelization to achieve further accelerations.

Datasets and Nomenclature

In this paper we use the following symbols to summarize the dimensions of genotype datasets:

- **N**, the number of subjects
- **M**, the number of genetic markers (variants)

- **P**, the number of phenotypes
- **C**, the number of covariates

Our motivating use case was a GWAS dataset from the COPDGene consortium [17] with N=6678, M=645,863, P=164, and C=7. The seven covariates were 5 principal components computed from the genotype matrix, age at study enrollment, and smoking status (in pack-years).

Test data sets for the contest were derived by sampling genotypes from a 1000 Genomes Project [18, 19] that included 1624 individuals from 8 populations with 100,000 markers per subject, then generating simulated phenotypes and covariates corresponding to the genotypes. For each test dataset, the 4 problem dimensions were uniformly sampled from these ranges: **N** 500-1500; **P** 3 and 50; **M** 1000-5000; and **C** 5 and 10. A genotype matrix (**N**x**M**) was then sampled from the 1624x10000 genotype matrix. For each phenotype, a liability value for each individual was computed assuming 0-5% of markers had non-zero phenotypic effects on a background of population effects, and the binary phenotype was set to 1 when the liability value was greater than zero, and zero otherwise. Finally, the covariate vectors were set to be the first **C** principal components of the normalized genotype matrix. Details of the genotype and phenotype simulation framework were included in the contest problem statement [20].

Compute Environments

Our routine compute environment consisted of a high-performance compute (HPC) cluster of about 2300 processors running the LSF job scheduler. Typical processors were Intel Xeon E7-8891 V2 (64 bit, 3.2 GHz), and nodes had 529GB RAM. Operating system was Red Hat Enterprise Linux 6.5.

The HPC environment was shared with multiple users across our organization, making it more difficult to capture consistent benchmark times. So additional testing, as noted in the Results section, was done on an Amazon Web Services (AWS) m4.4xlarge instance running the same operating system as HPC (Amazon machine instance ID = ami-6869aa05).

PLINK code profiling

PLINK 1.07 was profiled in the HPC environment to break down the computational costs of individual components of the logistic regression calculation. For this profiling, PLINK was compiled under gcc 4.1.2 with `-O2` and `-pg` options. Profiling was done using `gprof` 2.17.50.0.6, which reported the code call graph and fractions of time spent in specific code segments.

Logistic Regression Contest

To initialize our contest, a reference implementation of the PLINK 1.07 logistic regression code was created in a test harness suitable for contestants with no prior knowledge of genetics. The core regression code from PLINK was extracted and repackaged into a C++ class with a single public method called `computeAssociations()`. To eliminate the need for contestants to work with PLINK-specific file formats and data structures, the class was designed to read SNP data from a human-readable text file containing allele dosages, instead of PLINK's more compact but opaque `.bed` files.

A scoring metric that supported our goal of achieving both improved accuracy and speed was created and used as the sole metric to award prizes. The accuracy score component was calculated using the following procedure:

Contestants computed the M x P matrix of chi-square (Z) statistics, ordered in decreasing order of significance (i.e., decreasing order of Z^2).

The ranked list was compared to the reference (correct) result, computed using `computeAssociations()`.

The accuracy score was computed as the number of correct Z values computed (within 0.1% tolerance) before the first mistake.

A raw score for each test case was calculated as the accuracy score divided by a time penalty between 1.0 and 2.0 was defined by:

RAW_SCORE = ACCURACY_SCORE / (1 + max(TIME_SPENT, 100ms) / TIME_LIMIT)

where TIME_LIMIT is set to 100ms.

Finally, a scaled score for each test case was defined relative to the scores of other competitors:

SCALED_SCORE = RAW_SCORE / max(P, BEST)

where BEST is the best raw score achieved for that test case by any competitor and P is the number of phenotypes. The max(P, BEST) is intended to reduce score variance in the event of very difficult cases. The total score for a submission was the sum of the scaled scores over all test cases.

The contest was hosted as a 10-day marathon contest on TopCoder.com, an online programming competition web site [20]. Contestants were competing for a total of USD $10,000 in prize money where the first place contestant won $5000, second place won $2000, third place $1500 and fourth and fifth place received $1000, and $500 respectively.

Contract development with logistic regression contest winner

To enable PLINK users to take advantage of enhancements generated by crowdsourcing, we contracted for $2500 with the winner of the logistic regression contest to integrate his code with PLINK 1.07. Given the contestants' deep familiarity with his own code, this was an efficient way to integrate contest code into PLINK 1.07 with significantly less effort than would have been required by a third party. The integrated code module was then donated to the PLINK2 project.

Data input/initialization contest

Contestants were provided with the complete source code of PLINK 1.07 and a winning program from the logistic regression marathon contest. The focus of the contest was to revise the code to make the whole execution process faster by any means possible, but the contestants were directed to look specifically at the code that handled reading input data and initializing data structures (that is, the steps from reading input genotypes from disk, to the point where the in-memory data structures required for the contest algorithm to execute were created and loaded). Four test cases covering a range of problem sizes (**N**=100 individuals with **M**=5000, 50000, and 700000 markers; and **N**=6000 individuals with **M**=7000 markers) were provided to contestants. The largest test case was use to rank

performance. We required the solution to maintain correctness and provide at least a 2x speedup in processing times. The contest [21] was hosted on Topcoder.com as a first-to-finish race, meaning that the first solution satisfying all the requirements would be considered the winning solution and win the $300 prize.

Multithreading Contest

In addition to novel approaches to logistic regression, which we sought in the logistic regression contest, we also used crowdsourcing to acquire resources to do more "conventional" coding work, such as multithreading. Contestants were provided with source code created by winning contestants in the data input/initialization contest, and asked to multithread this code. The contest [22] was hosted on Topcoder.com. For testing, the number of threads was set to 4 and a successful solution required at least a 2-fold decrease in processing time. The prize consisted of two parts. The first contestant to achieve a 2x speed-up with multithreading won $600. The winning code was then made available to all other contestants. At the end of 2 weeks, the contestant with the submission with the fastest speed qualified for a prize of min($100*k* , $1000), where *k* is the additional fold-increase by which the code was accelerated, relative to the first winner's code. For example, if the first winner achieved a 2x speed-up, and a following contestant increased that to 3x, then k = 1 (3x - 2x = 1x).

Coarse-grained Parallelization

As a final step, we implemented coarse-grained (scatter-gather) parallelization using codes generated from the project. Multiple parallel processes running fast logistic regression code were executed using the Platform LSF load scheduling software on a high-performance compute cluster. The approach for coarse-grained parallelization was to run fast logistic regression on all input markers, identify those with significant association statistics, and then run an accelerated PLINK on the subset of markers with logistic association p-values less than a user-selected threshold to generate the final regression summary statistics.

Results

Summary of challenge/Problem formulation

Our goal was to dramatically accelerate association analysis for GWAS. We first collected use cases from genetic analysts to better understand the use of GWAS in our organization. The leading use case was the association of binary phenotypes with variants, using the logistic regression option in PLINK.

Code profiling in representative test datasets with covariates showed that PLINK 1.07's core logistic regression code (the `fitLM()` function) accounted for about 80% of run time, and data initialization and related overhead accounted for most of the remaining 20%. Thus, we decided to focus first on acceleration of the regression calculation. Given this breakdown of computing time, we anticipated that an infinitely fast logistic regression routine within the context of PLINK 1.07 would achieve an upper bound of 5-fold speedup for the overall end-to-end association calculation.

Logistic regression contest design and results

An OI and contest based crowdsourcing approach was used to develop innovative solutions. A number of steps were taken to make this contest more attractive to non-domain experts. First, our problem statement highlighted the genetics application of logistic regression, but stated the core challenge in generic mathematical terms. Example input data that was provided to the contestants was reduced to a numeric allele dosage format, eliminating any genetics-specific references to alleles or nucleotides, making it easier for solvers to apply their own diverse perspectives to create their own solutions [23] and to reduce barriers to entry for potential contestants with no domain knowledge of genetics. Second, as a baseline reference solution, contestants were presented with an isolated and simplified version of PLINK's `fitLM()` method, which contained PLINK's core logistic regression code. Extracting this function out of the ~98,000 lines of PLINK source code enabled contestants to rapidly understand and run the reference solution. Third, test data sets of the appropriate size were

provided, as described in the Methods section above.

A scoring mechanism was devised to reward computational efficiency and accuracy. Contestants were asked to increase performance while generating association test statistics that were identical (within 0.1%) to PLINK. The contestants were notified that it was acceptable to precisely compute association statistics for only the most significantly associated variant-phenotype pairs, if runtime was limiting, but did not provide any additional direction. All scores were displayed on a public real-time leaderboard. To prevent over-fitting, final scoring was calculated on 100 submission data sets that were not available to the contestants.

A ten day contest was hosted on TopCoder.com, an online programming competition website with an existing community of over 600,000 software developers that routinely compete to solve programming challenges [24]. The challenge attracted 320 participants, of whom 56 different contestants submitted 292 different versions of code. A prize pool of $10,000 was awarded to the top 5 contestants. It is estimated that 1120 person-hours were dedicated to this contest, making this a very cost-effective method.

The five highest-scoring contest solutions were compiled on our HPC environment and benchmarked against PLINK's core logistic regression code (i.e. the `computeAssociations()` reference solution), based on the average of 5 program runs. Strikingly, the 5 contest winners successfully accelerated logistic regression by 18- to 45-fold over the core logistic regression method from PLINK 1.07. **Table 1** shows run times for reference and contest codes. Given this impressive, order-of-magnitude speedup, we further explored the winning codes and the contest discussion-board narratives of the winning contestants to identify common themes and approaches used by the winning solutions.

We found the winning solutions incorporated new approaches that broadly fell into 2 families: numerical and computational changes, and new algorithmic ideas. In the following sections we summarize some of the approaches we observed.

Numerical and Computational: in this category, contestants modified elements of the logistic regression calculation to increase speed. One change was to replace the standard C `exp()` function with a faster variant that took advantage of single-instruction multiple data (SIMD) parallelism (see below). Another was to change the numerical method used to compute matrix decompositions in the Newton-Raphson iterations. Contestants replaced the singular-value decomposition (SVD) method used in the PLINK reference solution with Cholesky or QR methods [25] . In addition, opportunistic spot modifications were made to the code in at least one case. For example, in one matrix multiplication, a contestant re-ordered operations to change a [matrix]*[matrix]*[vector] operation into a [matrix]*[vector]*[matrix] operation, thus saving operations.

A key computational change that was made by multiple winners was to adapt calculations to use SIMD parallelism through streaming SIMD compiler extensions (SSE). This method takes advantage of modern CPU designs that can operate on multiple packed data elements in parallel. SIMD was adopted at various places in the logistic regression code, for example in the matrix decomposition steps. Adoption of SIMD appeared to be a major contributor to the observed speedups.

Algorithmic: An interesting algorithmic modification used by more than one winning contestant related to the initialization and execution of the Newton-Raphson iterations used to solve for the logistic coefficients. At the initialization of their solutions for each phenotype, contestants replaced PLINK's default initial values for the logistic coefficients with initial values determined by solving a covariate-only regression model for each phenotype. In practice, this often provided starting coefficient values that were closer to the final solution, especially when covariates accounted for much of the variance in phenotype. Contestants also observed that the first Newton iteration is computationally cheaper and can often produce a solution that is close to the correct result, and so they incorporated approaches that could use the result of that first Newton iteration to filter genotypes before executing more iterations on the subset of genotypes with strong associations.

Re-integration into PLINK

To create a code product that would be as portable as PLINK and could be directly donated back to the PLINK community, we contracted with the top-scoring contestant from the logistic regression contest to have him incorporate his accelerated logistic regression method tightly into PLINK 1.07, by replacing PLINK's `fitLM()` method with a drop-in replacement method that incorporated the faster code. One major advantage of our crowdsourcing efforts was to identify an expert with the skill set and the ability to solve our difficult problem. Given the winning contestants' established ability and familiarity with the code, the effort required to integrate the code was significantly less the effort that would have been required by a third party. This modified PLINK ran logistic association analyses 3.8-fold faster than PLINK 1.07 in the HPC environment, approaching our initial estimate of an upper bound of a 5-fold speed-up that could be achieved by accelerating the logistic regression component of the overall computational work. In the AWS environment, speedup was 7-fold (**Figure 2**), which we attributed to a different profile of data I/O versus computation cost in that environment, compared to HPC.

This modified PLINK, which we termed **`PLINK-FLR`** (fast logistic regression) was just as portable as PLINK 1.07 and thus well suited to donation back to the PLINK community. We provided **`PLINK-FLR`** to the PLINK2 project [26], and the logistic regression code was adopted in PLINK 1.9.

Additional code acceleration

In addition to donating this portable code back to the PLINK community, we anticipated that we could achieve substantial additional speedups, albeit possibly less portable, by further contest-based crowdsourcing. To explore this, we further developed the code generated by the logistic regression contest.

As mentioned above, for simplicity the code in the logistic regression contest took as input integer allele dosages in a text format. For real-world applications a more compact format such as the

PLINK .bed/.bim/.fam fileset is required. Therefore, we extracted from PLINK the code required to read .bed/.bim/.fam filesets, added methods to make the PLINK input compatible and integrated it with contestant codes, so the contestant codes could take as input native PLINK binary filesets. We also adjusted the output of contestant code so that p-values were generated (instead of the chi-squared statistics generated in the logistic regression contest). The end-to-end run time of the resulting program, called **C1**, on a test case with dimensions **N**=6678, **M**=645863, **P**=1, and **C**=7 was 9 times faster than the PLINK 1.07 in the AWS environment (**Figure 2**). This speedup was accounted for by a combination of a logistic regression algorithm that was ~35-fold faster than PLINK1.07, plus a relative reduction in data pre- and post-processing time, compared to PLINK 1.07. Importantly, part of the pre-processing time reduction was due to a change in the handling of missing genotypes. The code effectively presumes all genotypes are observed, and subjects with missing genotypes are not flagged and selectively excluded from regressions, as they are in PLINK1.07. Thus, the code is appropriate for application to datasets without missing genotypes. Datasets with missing genotypes could be analyzed after preprocessing the data to remove cases with missing genotypes, or imputing the missing genotypes.

Speedup of data initialization

Code **C1** still included a costly pre-processing step to transform the genotype matrix from the structures used within PLINK I/O code to the structures compatible with contestant code, so unsurprisingly data reading and initialization from PLINK `.bed` files emerged as a new rate-limiting step in the overall computation. We turned again to the open community to identify solutions to decrease the time required for this data initialization. The contestants were provided with code **C1**, and asked to revise the code however they saw fit but were directed to the rate limiting steps that included reading PLINK binary filesets and setup of initial genotype data structures prior to logistic regression.

A winner-take-all strategy was employed for this contest. This contest awarded the first

contestant to produce a solution that reduced the run-time by at least two-fold. This type of competition attracts fewer, but possibly more highly qualified participants since the question is specific and the prize pool is reduced in this scenario.

The winning solution accelerated the data initialization by modifying 'for' loop structures and vector initializations, removing some C++ vector operations, and eliminating an expensive transpose of the genotype matrix read from the `.bed` file. The end-to-end run time of the winning code (denoted **C2**) on a test dataset with **N**=6678, **M**=645863, **P**=1, and **C**=7 was decreased 13-fold compared to code **C1** (95 seconds vs. 1255 seconds) (**Figure 2**).

Multithreading

By design, GWAS analysis repeats the same type of calculation many times. Since modern operating systems and processors support multiple concurrent threads, GWAS analysis can take advantage of shared-memory parallel processing. We ran a contest on the TopCoder.com community to implement multithreading of our algorithm, awarding a prize to the first contestant who could achieve a 2-fold speedup of the baseline code from the second contest above. Code **C2** was provided to contestants along with three sample inputs and outputs to allow contestants to test locally if their modifications functioned and gave the correct result. A 16-day contest was run to identify a solution.

The winning code entry used OpenMP [27] to parallelize two components of the code. First, in the initialization of the genotype marker data matrix, prior to the logistic regression, a 'parallel for' construct was added to split work among threads. Second, the core logistic regression calculation for all markers was multi-threaded, to split the outer loop over the **M** markers among threads. The end-to-end run time of the winning code (denoted **C3**) running 4 threads on a test dataset with **N**=6678, **M**=645863, **P**=1, and **C**=7 was decreased 3.4-fold compared to code **C2** (28 seconds vs. 95 seconds) (**Figure 2**). This was consistent with our expectation of a relative speedup that approached the number of parallel threads.

Coarse-grained parallelization and PLINK-compatible output using HPC

Many investigators that run GWAS analysis, including our group, have access to high performance compute environments which use job management tools like IBM® Platform™ LSF® [28] or TORQUE [29] to do coarse-grained parallelization of calculations across many compute nodes. To help us process ever-larger genotype datasets, we wished to enable this type of scatter-gather parallelism. In addition, given that PLINK is a widely used community standard for GWAS analysis, we saw a substantial usability benefit in generating summary statistics in a format identical to PLINK 1.07. Our crowdsourced code did not provide that format as-is. Instead, it returned logistic regression p-values, without the additional summary statistics such as regression coefficients and confidence intervals that are provided by PLINK. We wished to generate PLINK-identical output reports, while avoiding the complexity of interfacing and co-compiling the contest-generated code into the PLINK 1.07 codebase.

To these ends, we established a "two-pass" analysis application. The crowd-sourced code from the multithreading contest described above (**C3**) was harnessed inside a script wrapper to submit parallel logistic regression jobs to the LSF scheduler. In the first analysis pass, **C3** was run in parallel and using the output p-values, markers were filtered according to a user-defined cutoff to exclude markers that had no significant association with the target phenotype (typically, this represents the vast majority of markers). In the second pass, the first round passing markers were submitted to **`PLINK-FLR`**, yielding standard PLINK logistic regression output files for that subset of markers that met the user's selected p-value cutoff value. Hence, the final statistical analysis output for the passing markers is in the standard PLINK 1.07 format. Since only a very small fraction of markers have significant associations in most GWAS, this "two pass" approach did not impose a notable performance penalty. This hybrid pipeline combining the crowd sourced code with PLINK was termed "**`mPLINK`**".

By distributing work across our HPC cluster, we expected to be able to rapidly process much

larger datasets than possible in the single-server AWS test environment. Hence, we tested the run time of **mPLINK** on three datasets with sizes ranging from 4 billion to 49 billion regressions using increasing numbers of processes ranging from 1 to 50. These jobs were executed on a shared cluster which contributed some variation to run times, but the main trends were clear (**Table 2**). Compared to PLINK1.07, we observed a dataset-size dependent speed increase ranging from 591- to 1450-fold in the HPC environment. At the smallest problem size (**N**=6678, **M**=645863), we observed sub-linear speedups as we went from 1-10 processes. At this problem size, with >10 processes, the overhead of scattering and gathering coarse-grained jobs dominated, limiting speedup to no more than 591X. For the two larger datasets, where a greater fraction of the time was spent in logistic regression routines, the relative speedup was larger (up to 1450X), although still sub-linear as the number of parallel processes was increased from 1-50, consistent with the presence of scatter-gather overheads. We attributed the observed overall speedup to the combination of the core logistic regression speedup (developed in **C1**), the data initialization changes (in **C2**), multithreading (in **C3**) and the application of coarse grained parallelization. In addition to the speedup we observed, by breaking up datasets across multiple large-memory compute nodes, HPC enabled us to run datasets including a size (**N**=7000, **M**=7,000,000) that would have exceeded the memory capacity of any widely available single-server environment. Note that we could have employed pre-processing methods like chromosome splitting to divide large datasets into smaller sizes that would not require access to large-memory environments. In our approach we used random-access into PLINK binary files to divide input datasets into flexibly-sized chunks for HPC processing, which was more convenient for us.

To verify the accuracy of our calculations, we compared p-values generated by the **C3** code to those generated by PLINK 1.07 (**Figure 3A**).

Real World Application

We applied **mPLINK** to one phenotype from the COPDGene consortium dataset [17] (**N**=6678, **M**=645,863). **Figure 3B** shows Manhattan plots from this dataset generated by PLINK 1.07 and

mPLINK with a user-defined p-value reporting cutoff of $p \leq 10^{-3}$. All of the significant markers identified by PLINK 1.07 on this dataset were also identified by **mPLINK**. A small number of markers that were close to the user defined cutoff were not returned by **mPLINK**, attributable primarily to the presence of missing values in this real-world dataset, and differences in convergence criteria between calculations.

The accelerated calculations in **mPLINK** provided us the ability to analyze and gain insight into more phenotypes in the COPDGene dataset. **mPLINK** was applied in the COPDGene study to analyze **P**=164 binary phenotypes at a rate of <1 minute per genotype, reducing analysis time from >20 days (estimated) to several hours, and allowing an exhaustive survey of all binary phenotypes in the dataset. The results were then clustered by phenotype and genotype to gain additional insights into the data (**Figure 3C**).

Discussion

The use of OI and crowdsourcing is becoming an important tool to address important and complex problems in biomedical research. Online platforms are now available that supply a community of solvers. The crowd provided by these platforms includes domain experts in a wide range of problem spaces. Reviewing the steps we took both before and after running contests allowed us to define some approaches and methods that we believe contributed to success for our project, and make some comparisons to more "traditional" approaches to the problem we tackled here.

Before the contest

Before beginning contests, the key steps we took were requirements-gathering, profiling of our "current state" solution, decomposition of the problem, creation of test sets, definition of our contest scoring method, and decision on contest type.

During requirements-gathering we interviewed GWAS practitioners in our institution to identify a relevant problem to solve, and confirm that the solution would be useful. This was followed by profiling of our current approach (PLINK 1.07) to understand what elements of the existing

GWAS analysis process were rate-limiting. Once the logistic regression was identified as first element to tackle, we decomposed the problem by extracting the logistic regression code from PLINK to create a minimal code that served as the contest baseline.

A critical step at this point was definition of the test data to be used to score the contest. It is essential that test data accurately reflect the "real-world" data that the code will see in all relevant respects. This is important particularly because contestants will naturally optimize their submission using the specific test data which they are provided. This can often lead to lack of generalizability if test data sets do not capture the diversity of real-world datasets. Finally, we devised a scoring system that rewarded our most desired outcomes of speed and accuracy.

The online platform we used offered different contest types. For the initial logistic regression contest we utilized a "Marathon Match". Marathon matches with significant prizes can attract skilled participants, and the competitive orientation of these contests can deliver innovative and extreme value outcome solutions. In contrast, for follow-on contests, we used "first-to-finish contests". These contests offered lower prizes and tended to attract fewer contestants but were effective at identifying crowd members who could execute specific coding tasks. Hence these contests were useful to provide capacity enhancement to our project team.

After the contest

After the contest, the key steps we took included evaluation of the solutions in our compute environment, review of the code, and merging, tracking, and supplementation of solutions over the course of multiple iterative contests. In general, these were standard scientific programming or software engineering activities that would occur in any software development project, but some elements were particularly salient in the crowdsourcing context.

Within a single marathon match, contest codes were written and sometimes optimized for the contest hardware/OS/compiler environment. An ideal setup would have ensured the contest environment was identical to the intended platform for final use of the code, but in practice that was

not always possible. For example, our HPC environment could not be provided to the contestants directly. Hence, we found that the final speed and performance characteristics of codes in our environment were not always identical to the contest ranking. For example, some fast logistic regression codes used specific tricks or data structures that were either limiting or not performance-enhancing in our environment. Hence, reviewing and benchmarking codes in our compute environment was essential.

Across multiple, serial competitions, it was necessary to select best codes from an initial contest, possibly supplement them (for example, interfacing to PLINK format input data), and then supply the modified codes as input to a subsequent contest. In at least one case, we found a participant in one contest might reverse or remove code elements that were desirable for the overall project, in order to maximize performance on their particular sub-problem. This behavior was not always easy to control through contest parameters. Given the possibility of multiple, potentially inconsistent code changes made at different stages by different authors, a source control system was invaluable for tracking codes over time. We used Apache Subversion [30] for this purpose.

Comparison to status-quo approach

In the absence of crowdsourcing, we would have executed this work as a software development project using either developers internal to our organization, or external contract workers. In our experience, the major benefit of the crowdsourcing approach for this project was the ability to rapidly recruit highly-skilled coders who could provide either innovative algorithmic enhancements or specific coding skillsets, at lower cost than our traditional approaches.

Conclusion

Using iterative competition based OI, we have substantially accelerated logistic regression for GWAS analysis. The accelerated logistic regression code was donated, incorporated and is currently available in the PLINK2 open-source project [26, 31] to make it broadly available to the

computational biology community, where it can enable the analysis of increasingly complex phenotype-genotype datasets.

Availability and requirements

• Project Name: GWAS logistic regression project

• Project Page: https://github.com/hillan141/gwas-speedup

• Operating System: Linux

• Programming Language: C, C++

• Other Requirements: Codes have been tested on Red Hat Enterprise Linux 6 with gcc 4.4.7, 32 GB RAM.

• License: GPLv2

Availability of supporting data

Snapshots of the source code of the software are available in the *GigaScience* GigaDB repository [32].

Ethics approval and consent to participate

Not applicable

Competing interests

The authors declare that they have no competing interests.

Author's contributions

SAJ, AH, RBB, PRL, IK and KL designed the project. PP, JS developed algorithms. AH implemented and tested the algorithms. EG provided guidance. AH and SAJ drafted the manuscript. All authors edited, read and approved the final manuscript

Acknowledgements

We would like to thank the Pfizer Business Technology High Performance Compute group for their assistance with this project. We would like to thank Sally John and Christoph Brockel for providing guidance and thoughtful discussions. We also like to thank the members of the Topcoder community for participation in our contest and in particular the winning contestants, doudouille, allegro, zaq1xsw2tktk, syg96, venco, and klo86min.

References

1. Altshuler D, Daly MJ, Lander ES: **Genetic mapping in human disease**. *Science* 2008, **322**(5903):881-888.
2. Frazer KA, Murray SS, Schork NJ, Topol EJ: **Human genetic variation and its contribution to complex traits**. *Nature reviews Genetics* 2009, **10**(4):241-251.
3. Visscher PM, Brown MA, McCarthy MI, Yang J: **Five years of GWAS discovery**. *American journal of human genetics* 2012, **90**(1):7-24.
4. Witte JS: **Genome-wide association studies and beyond**. *Annual review of public health* 2010, **31**:9-20 24 p following 20.
5. Purcell S, Neale B, Todd-Brown K, Thomas L, Ferreira MA, Bender D, Maller J, Sklar P, de Bakker PI, Daly MJ *et al*: **PLINK: a tool set for whole-genome association and population-based linkage analyses**. *American journal of human genetics* 2007, **81**(3):559-575.
6. Prabhu S, Pe'er I: **Ultrafast genome-wide scan for SNP–SNP interactions in common complex disease**. *Genome Res* 2012, **22**(11):2230-2240.
7. Kam-Thong T, Czamara D, Tsuda K, Borgwardt K, Lewis CM, Erhardt-Lehmann A, Hemmer B, Rieckmann P, Daake M, Weber F *et al*: **EPIBLASTER-fast exhaustive two-locus epistasis detection strategy using graphical processing units**. *Eur J Hum Genet* 2011, **19**(4):465-471.
8. Schüpbach T, Xenarios I, Bergmann S, Kapur K: **FastEpistasis: a high performance computing solution for quantitative trait epistasis**. *Bioinformatics* 2010, **26**(11):1468-1469.
9. Zhang X, Zou F, Wang W: **FastChi: an efficient algorithm for analyzing gene-gene interactions**. In: *Pacific Symposium on Biocomputing Pacific Symposium on Biocomputing: 2009*. NIH Public Access: 528.
10. Ma L, Runesha HB, Dvorkin D, Garbe JR, Da Y: **Parallel and serial computing tools for testing single-locus and epistatic SNP effects of quantitative traits in genome-wide association studies**. *BMC Bioinformatics* 2008, **9**:315-315.
11. Good BM, Su AI: **Crowdsourcing for bioinformatics**. *Bioinformatics* 2013, **29**(16):1925-1933.
12. Lakhani KR, Boudreau KJ, Loh PR, Backstrom L, Baldwin C, Lonstein E, Lydon M, MacCormack A, Arnaout RA, Guinan EC: **Prize-based contests can provide solutions to computational biology problems**. *Nature biotechnology* 2013, **31**(2):108-111.
13. Leiter A, Sablinski T, Diefenbach M, Foster M, Greenberg A, Holland J, Oh WK, Galsky MD: **Use of crowdsourcing for cancer clinical trial development**. *Journal of the National Cancer Institute* 2014, **106**(10).
14. Marbach D, Costello JC, Kuffner R, Vega NM, Prill RJ, Camacho DM, Allison KR, Consortium D, Kellis M, Collins JJ *et al*: **Wisdom of crowds for robust gene network inference**. *Nature methods* 2012, **9**(8):796-804.
15. Costello JC, Heiser LM, Georgii E, Gonen M, Menden MP, Wang NJ, Bansal M, Ammad-ud-

din M, Hintsanen P, Khan SA *et al*: **A community effort to assess and improve drug sensitivity prediction algorithms**. *Nature biotechnology* 2014, **32**(12):1202-1212.
16. Holland RC, Lynch N: **Sequence squeeze: an open contest for sequence compression**. *GigaScience* 2013, **2**(1):5.
17. Cho MH, McDonald ML, Zhou X, Mattheisen M, Castaldi PJ, Hersh CP, Demeo DL, Sylvia JS, Ziniti J, Laird NM *et al*: **Risk loci for chronic obstructive pulmonary disease: a genome-wide association study and meta-analysis**. *The Lancet Respiratory medicine* 2014, **2**(3):214-225.
18. Genomes Project C, Abecasis GR, Auton A, Brooks LD, DePristo MA, Durbin RM, Handsaker RE, Kang HM, Marth GT, McVean GA: **An integrated map of genetic variation from 1,092 human genomes**. *Nature* 2012, **491**(7422):56-65.
19. Genomes Project C, Abecasis GR, Altshuler D, Auton A, Brooks LD, Durbin RM, Gibbs RA, Hurles ME, McVean GA: **A map of human genome variation from population-scale sequencing**. *Nature* 2010, **467**(7319):1061-1073.
20. **TopCoder GWASSpeedup Marathon Match Problem Statement** [http://community.topcoder.com/longcontest/?module=ViewProblemStatement&rd=15637&pm=12525]
21. **GWAS Speedup Integration Bug Race-1** [https://apps.topcoder.com/bugs/browse/BUGR-9401]
22. **GWAS Speedup Integration Bug Race-2** [https://apps.topcoder.com/bugs/browse/BUGR-9693]
23. Hong L, Page SE: **Groups of diverse problem solvers can outperform groups of high-ability problem solvers**. *Proceedings of the National Academy of Sciences of the United States of America* 2004, **101**(46):16385-16389.
24. Eiben CB, Siegel JB, Bale JB, Cooper S, Khatib F, Shen BW, Players F, Stoddard BL, Popovic Z, Baker D: **Increased Diels-Alderase activity through backbone remodeling guided by Foldit players**. *Nature biotechnology* 2012, **30**(2):190-192.
25. Press WH, Teukolsky SA, Vetterling WT, Flannery BP: **Numerical Recipies in Fortran**, 2nd edn. Cambridge: Cambridge University Press; 1992.
26. Chang CC, Chow CC, Tellier L, Vattikuti S, Purcell SM, Lee JJ: **Second-generation PLINK: rising to the challenge of larger and richer datasets**. *Gigascience* 2015, **4**(7).
27. **The OpenMP API specification for parallel programming** [http://openmp.org/wp]
28. **IBM Platform Computing LSF products** [http://www.ibm.com/systems/technicalcomputing/platformcomputing/products/lsf/]
29. **TORQUE Resource Manager** [http://www.adaptivecomputing.com/products/open-source/torque/]
30. **Apache Subversion** [https://subversion.apache.org/]
31. **PLINK2 Project home page** [https://www.cog-genomics.org/plink2]
32. Andrew Hill; Po-Ru Loh; Ragu B Bharadwaj; Pascal Pons; Jingbo Shang; Eva Guinan; Karim Lakhani;Iain Kilty; Scott A Jelinsky. (2016): Supporting materials from “Stepwise Distributed Open Innovation Contests for Software Development - Acceleration of Genome-Wide Association Analysis”. GigaScience Database. http://doi.org/10.5524/100264

Figure Legends

Figure 1: Iterative open source contests to accelerate logistic regression for GWAS analysis

Workflow and code outputs of our project. First, a 10 day marathon crowd sourcing competition was hosted to accelerate the logistic regression code from PLINK 1.07, yielding code **C1**. Accelerated logistic regression code was integrated back into PLINK 1.07, yielding code **PLINK-FLR.** The logistic regression elements were donated and integrated into the PLINK2 project. A first to finish contest was run to speed up data initialization in the C1 code, yielding code C2. Another first to finish contest was run to multithread the C2 code, yielding code C3. C3 was then combined with coarse-grained HPC parallelization and PLINK-FLR, yielding **mPLINK**.

Figure 2 : Run time of codes. Run times of a test case with dimensions **N**=6678, **M**=645863, **C**=7, **P**=1 were determined. Shown are run times for PLINK 1.07 (P1.07) , **PLINK-FLR(**P-FLR**)**, **C1**, **C2**, **C3** and **mPLINK** run with 10 processors. Values above the column represent run times in seconds. See text for detailed description of codes.

Figure 3: GWAS analysis results.

(A) Scatter plot comparison of –log10 p-values for a synthetic test case with dimensions **N**=6678, **M**=645863, **C**=7, **P**=1 and no missing values. PLINK 1.07 output was compared to the output of **C3**. 97% of P-values computed by **C3** are within a 0.1% relative tolerance of reference p-values from PLINK 1.07. (B) Manhattan plots for real-world test case from COPDGene study with same dimensions as (A). Top panel: all P-values as computed by **PLINK**. P-values above user-set threshold of p=0.001 are colored red. Bottom panel: Second-pass (final) **mPLINK** P-values for markers meeting the p=0.001 threshold in the first round. A small number of markers fall below the p=0.001 cutoff due to differences in missing value handling and convergence criteria in **C3**, versus **PLINK-FLR**. Compute time was approximately 29 seconds for **mPLINK**, Compared to 4.7 hours for

PLINK 1.07. (C) Two-way clustering of SNPs and phenotypes according to SNP-phenotype association p-values. 164 binary phenotypes from the COPDGene study were associated against each of the **M**=645,863 SNPs in the study. Results were filtered to variants which had any logistic association p-value <4.81e-9 (i.e. a Bonferroni adjusted p-value of 0.05, for N=645,683 SNPS and P=164 traits).

Tables

Table 1: Acceleration of logistic regression.

**Code**	**n (# replicated runs)**	**Avg Time (sec)**	**SD Time (sec)**	**Fold-speedup vs PLINK 1.07**
PLINK 1.07 (--assoc)	*1*	*88*	*NA*	***NA***
PLINK 1.07 (logistic regression only)	5	68.8	14.58	**1**
LRC4	5	1.5	0.03	**45**
LRC5	5	1.9	0.05	**36**
LRC1	5	2.3	0.48	**30**
LRC3	5	3.8	1.17	**18**
LRC2	5	3.9	0.06	**18**

All results are on a test set with **N**=6000, **M**=7000, **P**=1, and **C**=5, in the HPC environment where N is the number of subjects and M is the number of genetic markers (variants), P, is the number of phenotypes and C is the number of covariates. First row of table indicates the end-to-end run time of PLINK 1.07, for context. Subsequent lines indicate run times of isolated logistic regression routines.

Table 2: mPLINK wall-clock runtimes (seconds).

	**Test Case**		
	**N6678 X M645863**	**N6678 X M3200000**	**N7000 X M7000000**
M*N	4,313,073,114	21,369,600,000	49,000,000,000
**Software run**			
PLINK-1.07	**17,146**	**70,617**	**172,602**
mPLINK (1 process)	**94**	NA (RAM)*	NA (RAM)*
mPLINK (5 process)	**34**	**109**	**281**
mPLINK (10 process)	**29**	**111**	**199**
mPLINK (50 process)	**39**	**60**	**119**
Max speedup (fold)	**591X**	**1177X**	**1450X**

*NA(RAM) signifies that the dataset was too large to load into memory and therefore was not calculated

Note: N refers to the number of subjects; **M** is the number of genetic markers (variants).

 Click here to download Figure Figure1v1.pdf

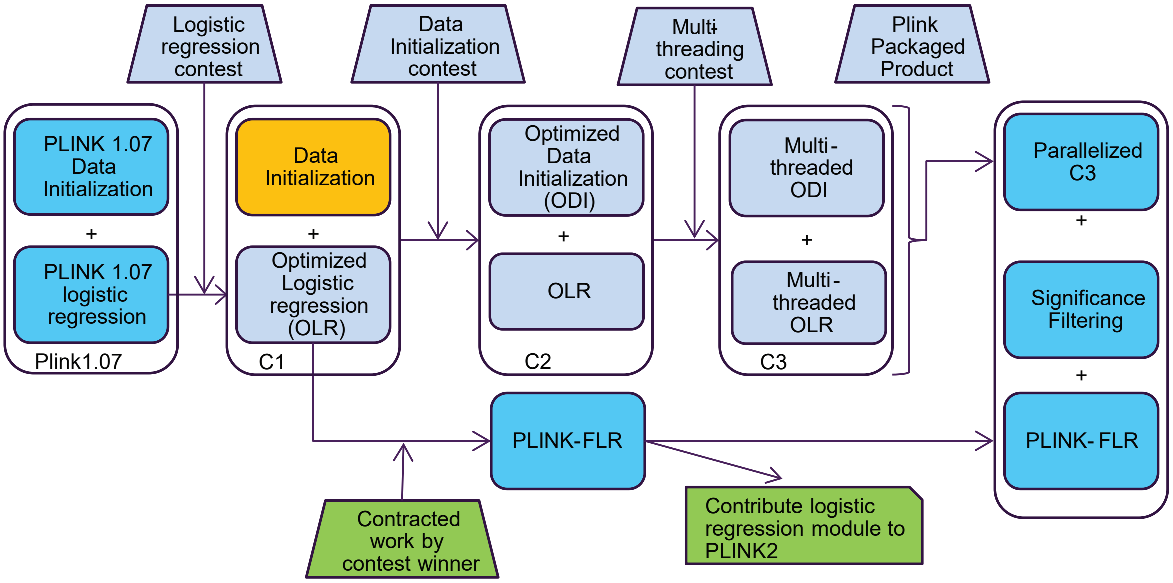

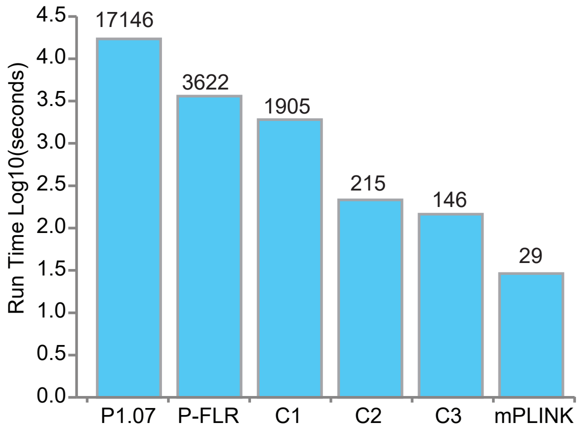
Run Time Log10(seconds)
4.5
4.0
3.5
3.0
2.5
2.0
1.5
1.0
0.5
0.0
17146
3622
1905
215
146
29
P1.07
P-FLR
C1
C2
C3
mPLINK

Figure3

Click here to download Figure Figure3v2.pdf

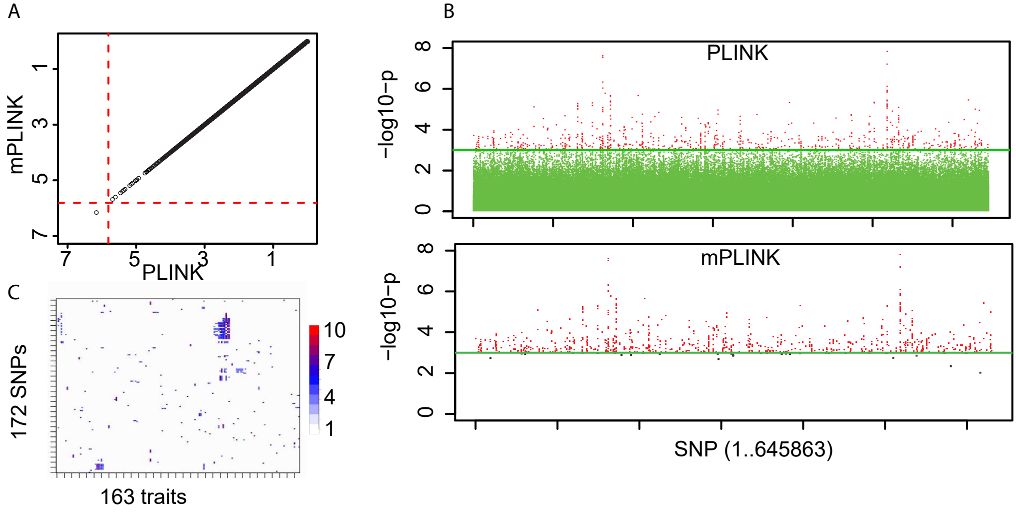

Supplement: GIGA-D-16-00109_Revision_1.pdf [file gix009_GIGA-D-16-00109_Revision_1.pdf]
