# Supplementary material for: Stepwise Distributed Open Innovation Contests for Software Development: Acceleration of Genome-Wide Association Analysis
Source: Gigascience. 2017 Feb 28;6(5):1–10. doi: 10.1093/gigascience/gix009 (PMC5467032; doi:10.1093/gigascience/gix009)
Supplement: Response_to_Reviewer_Comments_Original_Submission.pdf [file gix009_Response_to_Reviewer_Comments_Original_Submission.pdf]

GIGA-D-16-00109
Stepwise Distributed Open Innovation Contests for Software Development - Acceleration of Genome-Wide Association Analysis Andrew Hill, Ph.D.; Po-Ru Loh, Ph.D.; Ragu Bharadwaj, Ph.D.; Pascal Pons, Ph.D.; Jingbo Shang; Eva Guinan, MD; Karim Lakhani, Ph.D.; Iain Kilty, Ph.D.; Scott A Jelinsky, Ph.D.
GigaScience

Dear Dr Scott Edmunds, Ph.D.

Thank you for consideration of our manuscript "Stepwise Distributed Open Innovation Contests for Software Development - Acceleration of Genome-Wide Association Analysis" (GIGA-D-16-00109). We appreciate the comments and the effort of the reviewers. Below please find our specific responses to the reviewers' comments and concerns.

Reviewer 1 was "was not completely sure if incorporation in project meant available to users". Our accelerated logistic regression has been incorporated into the PLINK2 project and is currently available to users of PLINK 1.9beta. We have modified the text to read "The accelerated logistic regression code was donated, incorporated and is currently available in the PLINK2 open-source project"

Reviewer 1 commented that contestants not only compete for "Cash prizes but wider recognition is maybe another driver here". We completely agree and have added the following sentence to reflect different motivations. "While contestants generally compete for monetary prizes, there are other motivating factors including peer recognition, skill building, self –affirmation and perceived enjoyment that attract competitors".

Reviewer 1 commented that "Was any analysis done on the level of accuracy needed for comparison? For this type of process would 99.99 be adequate for its utility?". First we apologize to the reviewer as there was a typo in the manuscripts and we tested for accuracy to 99.9%. The "results" that are required to be correct to 0.1% are chi-square association test statistics. These statistics are generally only reported to 1 or 2 significant figures (e.g., in the GWAS catalog), so 0.1% error is adequate. We have modified the text to now read "Contestants were asked to increase performance while generating association test statistics that were identical (within 0.1%) to PLINK."

Reviewer 1 commented that "In tables, it might be worth reiterating what N, M, P are?". We agree and have made the changes:
• In Table 1, the definitions of N, M, P, and C are now reiterated in the caption
• In Table 2, the definitions of N and M are now reiterated in the caption

Reviewer 1 commented that "Fig 3 seems not well produced in the pdf I have". We have made some modifications to figure 3 and will work with the copy editor to be sure that the figure is well reproduced in the published version

Reviewer 1 recommended another manuscript on OI competition. We thank the reviewer for bringing this manuscript to our attention and have added this as a reference.

Reviewer 2 commented that on bottom of page 15 “It would be more accurate to say that mPLINK/HPC makes it more convenient to analyze such [large] datasets”. This is an excellent point and we have modified our manuscript and added the following sentences to reflect this. “Note that we could have employed pre-processing methods like chromosome splitting to divide large datasets into smaller sizes that would not require access to large-memory environments. In our approach we used random-access into PLINK binary files to divide input datasets into flexibly-sized chunks for HPC processing, which was more convenient for us.”

Reviewer 2 recommended “To make the quality of mPLINK more transparent, a few data points re: how wall-clock time decreases with the number of processes may be in order”. To address this we have:

• Updated the header of Table 2 to indicate more clearly that the run times shown are wall-clock times

• At page 16 lines 1-11, elaborated on how wall-clock time decreases with the number of processes to better explain the run times recorded in Table 2. The new text reads: “we tested the run time of mPLINK on three datasets with sizes ranging from 4 billion to 49 billion regressions using increasing numbers of processes ranging from 1 to 50. These jobs were executed on a shared cluster which contributed some variation to run times, but the main trends were clear (Table 2). Compared to PLINK1.07, we observed a dataset-size dependent speed increase ranging from 591- to 1450-fold in the HPC environment. At the smallest problem size (N=6678, M=645863), we observed sub-linear speedups as we went from 1-10 processes. At this problem size, with >10 processes, the overhead of scattering and gathering coarse-grained jobs dominated, limiting speedup to no more than 591X. For the two larger datasets, where a greater fraction of the time was spent in logistic regression routines, the relative speedup was larger (up to 1450X), although still sub-linear as the number of parallel processes was increased from 1-50, consistent with the presence of scatter-gather overheads.”

We thank Reviewer 2 for identifying some typos. The following has been updated:

* First paragraph of page 7: "Where" change to “where”.
* Extraneous right-parenthesis at end of second sentence removed.
* Third paragraph on page 9: "(within 0.01%)" changed to "(within 0.1%)".
* End of page 10: "in at least once case" changed to "in at least one case"
* First paragraph of page 11: "adopt calculations" changed to "adapt calculations"

Reviewer #3 commented that the “It is still unclear how much was the cost of the contract, and how difficult the integration was”. To address this we have modified the text in the methods section on page 7 to read “we contracted for $2500 with the winner of the logistic regression contest to integrate his code with PLINK 1.07. Given the contestants’ deep familiarity with his own code, this was an efficient way to integrate contest code into PLINK 1.07 with significantly less effort than would have been required by a third party”.

Reviewer 3 raised the question and recommendation: “What does the data initialization refer to in this paper? This should be clarified at the beginning”. To clarify this, at page 7, when data initialization is first referred to in the Methods section, we have added the explanation: “and initializing data structures (that is, the steps from reading input genotypes from disk, to the point

where the in-memory data structures required for the contest algorithm to execute were created and loaded).”

We have addresses reviewer 3’s minor concerns below.
1. Page 4 line 19: Life science has been changed to life science?
2. Page 5 line 26: What is the heritability for simulated phenotype? The heritability (of disease liability) was sampled uniformly between 0 and 1. The phenotype model included population stratification and noise. The details of the simulation framework that generated the phenotypes are given in our reference [20] (here). Conceivably some approximations of logistic regression might work well for estimating strong associations but work poorly for weak associations. For this reason the simulation framework was set up to explore a range of genetic architectures. The manuscript has been updated at page 5 line 15 to clarify that the details of the simulation framework are available at reference [20].
3. Page 6 and line 26: How accuracy and speed are combined to determine the prize? The final scoring metric is the sum of SCALED_SCORE per test case. This is defined on Page 6 line 53
4. Page 6 line 50: What do 1 and 2 refer t? The method used is also defined on line 53. We have modified the test to 1.0 and 2.0 to make it clear that they refer to enumerated items
5. Page 7 line 9: ")" has been removed
6. Page 7 line 6: Please explain why to choose maximum between P and Best. We needed to safeguard against the possibility of test cases in which all submissions achieved very low scores (e.g., failing to identify any top hits, which would give BEST=0, or succeeding in only identifying a small number of hits, which would cause BEST to be very small). Either way, using simply SCALED_SCORE = RAW_SCORE / BEST would cause high variance if the denominator was small. Therefore the max(P, BEST) is intended to reduce score variance in the event of very difficult cases.
7. Page 7 line 26: Is there additional cost to contract code from the winner? As discussed above, we have modified the text to include this information
8. Page 8 line 16: Does "fastest" mean the first submission or the submission with the fastest speed? Fastest refers to the submission with the fastest speed. We have modified the text to read “…the contestant with the submission with the fastest speed qualified for a prize…”
9. Page 9 line 60: Did contestants know the final score would be calculated on the 100 submission datasets that are not available to them? Yes. The contest problem statement (reference [20] in the manuscript, available here) stated that there were 100 full submission cases, and that the total score for a submission would be the sum of the scaled scores over all test cases.
10. Page 10 line 10: What are the average and maximum hour participants spend? We have estimated this based on the number and timing of the individual submissions. It is likely an underestimate as contestants could run and test locally. While this would be very interesting to determine it is not clear or obvious how to determine the exact number of hours per participant.
11. Page 17 line 9: Does value mean probability? No value means useful. We have modified the text and changed “would have value” to read “would be useful” to make this more clear.
12. Page 19 line 51: Are these user names recognizable for public or anywhere? These are all members of the Topcoder community and there profiles are publically available at TopCoder.com
13. Figure 1: Figure 1 has been updated to avoid overlaps between arrows and lines
14. Figure2: The figure legend has been updated to reflect that 10 processors were used in

mPLINK is 10 and to clarify that the numbers above the columns refers to the run time in seconds.

15. Figure 2: The figure has been updated to include only one decimal place
16. Figure 3A: Figure 3 has been updated to make it more readable
17. Figure 3: Figure 3 has been updated to make it more readable
