# Supplementary material for: Stepwise Distributed Open Innovation Contests for Software Development: Acceleration of Genome-Wide Association Analysis
Source: Gigascience. 2017 Feb 28;6(5):1–10. doi: 10.1093/gigascience/gix009 (PMC5467032; doi:10.1093/gigascience/gix009)
Supplement: Reviewer_2_Report_(Original_Submission).pdf [file gix009_Reviewer_2_Report_(Original_Submission).pdf]

Reviewer Report

Title: "Stepwise Distributed Open Innovation Contests for Software Development - Acceleration of Genome-Wide Association Analysis"

Version: Original Submission **Date:** 10/17/2016

Reviewer name: Chris Chang

Reviewer Comments to Author:

This is a useful description of how a frequently-used open source module was improved by multiple orders of magnitude on a reasonable budget without needing a domain expert on the team. Furthermore, as a domain expert who used the resulting code upgrades, I can confirm that the lion's share of the prize money drove innovation which went beyond what nearly all domain experts would have achieved in a reasonable amount of time. Logistic regression is just messy enough that a single expert is unlikely to have all the answers.

I just have a few minor revisions to recommend (page numbers refer to the .docx):

1. (bottom of page 15) "by breaking up datasets across multiple large-memory compute nodes, HPC enabled us to run datasets including a size (N=7000, M=7,000,000) that would have exceeded the memory capacity of any widely available single-server environment.": while this is technically true with naive use of PLINK 1.07, which unnecessarily loads the entire genotype matrix into memory before proceeding with the logistic regression, it's not a meaningful advantage of HPC. Chromosome-splitting is a simple workaround frequently employed by researchers without access to HPC environments. It would be more accurate to say that mPLINK/HPC makes it more convenient to analyze such datasets.

2. To make the quality of mPLINK more transparent, a few data points re: how wall-clock time decreases with the number of processes may be in order.

3. And some typos:

* First paragraph of page 7: initial "Where" probably should be lowercased. Extraneous right-parenthesis at end of second sentence.

* Third paragraph on page 9: Looking at the GWASSpeedup problem statement, "(within 0.01%)" should be "(within 0.1%)".

* End of page 10: "in at least once case" -> "in at least one case"

* First paragraph of page 11: "adopt calculations" -> "adapt calculations"

Methods

Are the methods appropriate to the aims of the study, are they well described, and are necessary controls included? Yes

Conclusions

Are the conclusions adequately supported by the data shown? Yes

Reporting Standards

Does the manuscript adhere to the journal's guidelines on minimum standards of reporting? Yes

Statistics

Are you able to assess all statistics in the manuscript, including the appropriateness of statistical tests used? Yes, and I have assessed the statistics in my report.

Quality of Written English

Please indicate the quality of language in the manuscript: Acceptable

Declaration of Competing Interests

Please complete a declaration of competing interests, considering the following questions:

- Have you in the past five years received reimbursements, fees, funding, or salary from an organisation that may in any way gain or lose financially from the publication of this manuscript, either now or in the future?
- Do you hold any stocks or shares in an organisation that may in any way gain or lose financially from the publication of this manuscript, either now or in the future?
- Do you hold or are you currently applying for any patents relating to the content of the manuscript?
- Have you received reimbursements, fees, funding, or salary from an organization that holds or has applied for patents relating to the content of the manuscript?
- Do you have any other financial competing interests?
- Do you have any non-financial competing interests in relation to this paper?

If you can answer no to all of the above, write 'I declare that I have no competing interests' below. If your reply is yes to any, please give details below.

I declare that I have no competing interests.

I agree to the open peer review policy of the journal. I understand that my name will be included on my report to the authors and, if the manuscript is accepted for publication, my named report including any attachments I upload will be posted on the website along with the authors' responses. I agree for my report to be made available under an Open Access Creative Commons CC-BY license (http://creativecommons.org/licenses/by/4.0/). I understand that any comments which I do not wish to

be included in my named report can be included as confidential comments to the editors, which will not be published.

I agree to the open peer review policy of the journal

To further support our reviewers, we have joined with Publons, where you can gain additional credit to further highlight your hard work (see: https://publons.com/journal/530/gigascience). On publication of this paper, your review will be automatically added to Publons, you can then choose whether or not to claim your Publons credit. I understand this statement.

Yes
