# Supplementary material for: Stepwise Distributed Open Innovation Contests for Software Development: Acceleration of Genome-Wide Association Analysis
Source: Gigascience. 2017 Feb 28;6(5):1–10. doi: 10.1093/gigascience/gix009 (PMC5467032; doi:10.1093/gigascience/gix009)
Supplement: Reviewer_3_Report_(Original_Submission).pdf [file gix009_Reviewer_3_Report_(Original_Submission).pdf]

Reviewer Report

Title: "Stepwise Distributed Open Innovation Contests for Software Development - Acceleration of Genome-Wide Association Analysis"

Version: Original Submission **Date:** 10/25/2016

Reviewer name: Zhiwu Zhang

Reviewer Comments to Author:

PLINK is the most popular software for GWAS. This manuscript described the endeavor of speeding up for 591-fold by using crowdsourcing, a new path for software development. The experiences are valuable to inspire other software development and make full usage of intelligent workforce.

Major concerns

1. The manuscript did mentioned winners were contracted to integrate the winning source code to PLINK2 project. It is still unclear how much was the cost of the contract, and how difficult the integration was. These are critical to make practical usage of crowdsourcing.

2. What does the data initialization refer to in this paper? This should be clarified at the beginning.

Minor concerns

1. Page 4 line 19: Life science or live science?

2. Page 5 line 26: What is the heritability for simulated phenotype?

3. Page 6 and line 26: How accuracy and speed are combined to determine the prize?

4. Page 6 line 50: What do 1 and 2 refer t?

5. Page 7 line 9: Remove ")"

6. Page 7 line 6: Please explain why to choose maximum between P and Best.

7. Page 7 line 26: Is there additional cost to contract code from the winner?

8. Page 8 line 16: Does "fastest" mean the first submission or the submission with the fastest speed?

9. Page 9 line 60: Did contestants know the final score would be calculated on the 100 submission datasets that are not available to them?

10. Page 10 line 10: What are the average and maximum hour participants spend?

11. Page 17 line 9: Does value mean probability?

12. Page 19 line 51: Are these user names recognizable for public or anywhere?

13. Figure 1: Try to avoid overlaps between arrows and lines (e.g. arrow to PLINK-FLR), or displaying arrows in half (e.g. arrow to "Contribute logistic").

14. Figure2: What's number of processors used in mPLINK? It seems not mentioned in the paper. And What do the values mean which labeled above the columns?

15. Figure 2: Only one decimal is needed for labeling y axis if interval is .5, e.g., 4.0 and 4.5.

16. Figure 3A: Dots are displayed well, especially for -log(P) > 7. Suggest bigger unfilled circles.

17. Figure 3: Label plot on bottom right (Manhattan plot by mPLINK) as "D".

Methods

Are the methods appropriate to the aims of the study, are they well described, and are necessary controls included? Yes

Conclusions

Are the conclusions adequately supported by the data shown? Yes

Reporting Standards

Does the manuscript adhere to the journal's guidelines on minimum standards of reporting? Yes

Statistics

Are you able to assess all statistics in the manuscript, including the appropriateness of statistical tests used? There are no statistics in the manuscript.

Quality of Written English

Please indicate the quality of language in the manuscript: Acceptable

Declaration of Competing Interests

Please complete a declaration of competing interests, considering the following questions:

- Have you in the past five years received reimbursements, fees, funding, or salary from an organisation that may in any way gain or lose financially from the publication of this manuscript, either now or in the future?
- Do you hold any stocks or shares in an organisation that may in any way gain or lose financially from the publication of this manuscript, either now or in the future?

- Do you hold or are you currently applying for any patents relating to the content of the manuscript?
- Have you received reimbursements, fees, funding, or salary from an organization that holds or has applied for patents relating to the content of the manuscript?
- Do you have any other financial competing interests?
- Do you have any non-financial competing interests in relation to this paper?

If you can answer no to all of the above, write 'I declare that I have no competing interests' below. If your reply is yes to any, please give details below.

I declare that I have no competing interest.

I agree to the open peer review policy of the journal. I understand that my name will be included on my report to the authors and, if the manuscript is accepted for publication, my named report including any attachments I upload will be posted on the website along with the authors' responses. I agree for my report to be made available under an Open Access Creative Commons CC-BY license (http://creativecommons.org/licenses/by/4.0/). I understand that any comments which I do not wish to be included in my named report can be included as confidential comments to the editors, which will not be published.

I agree to the open peer review policy of the journal

To further support our reviewers, we have joined with Publons, where you can gain additional credit to further highlight your hard work (see: https://publons.com/journal/530/gigascience). On publication of this paper, your review will be automatically added to Publons, you can then choose whether or not to claim your Publons credit. I understand this statement.

Yes
